# A systematic review of handover actions in human dyads

**DOI:** 10.3389/fpsyg.2023.1147296

**Published:** 2023-05-04

**Authors:** Lena Kopnarski, Julian Rudisch, Claudia Voelcker-Rehage

**Affiliations:** Department of Neuromotor Behavior and Exercise, Institute of Sport and Exercise Sciences, University of Münster, Münster, Germany

**Keywords:** object handover, kinematics, grip force, joint action, human dyads

## Abstract

**Introduction:**

Handover actions are joint actions in which an object is passed from one actor to another. In order to carry out a smooth handover action, precise coordination of both actors’ movements is of critical importance. This requires the synchronization of both the kinematics of the reaching movement and the grip forces of the two actors during the interaction. Psychologists, for example, may be interested in studying handover actions in order to identify the cognitive mechanisms underlying the interaction of two partners. In addition, robotic engineers may utilize insights from sensorimotor information processing in human handover as models for the design controllers in robots in hybrid (human-robot) interaction scenarios. To date, there is little knowledge transfer between researchers in different disciplines and no common framework or language for the study of handover actions.

**Methods:**

For this reason, we systematically reviewed the literature on human-human handover actions in which at least one of the two types of behavioral data, kinematics or grip force, was measured.

**Results:**

Nine relevant studies were identified. The different methodologies and results of the individual studies are here described and contextualized.

**Discussion:**

Based on these results, a common framework is suggested that, provides a distinct and straightforward language and systematics for use in future studies. We suggest to term the actors as *giver* and *receiver*, as well as to subdivide the whole action into four phases: (1) *Reach and grasp*, (2) *object transport*, (3) *object transfer*, and (4) *end of handover* to comprehensively and clearly describe the handover action. The framework aims to foster the necessary exchange between different scientific disciplines to promote research on handover actions. Overall, the results support the assumption that givers adapt their executions according to the receiver’s intentions, that the start of the release of the object is processed feedforward and that the release process is feedback-controlled in the transfer phase. We identified the action planning of the receiver as a research gap.

## 1. Introduction

The handing over of a salt shaker at dinner or a surgical tool from a nurse to a doctor are examples of handover actions that take place as a matter of course in everyday life. A handover action is effective when both actors achieve a smooth transfer of an object from one person to the other. A high degree of intrapersonal coordination (the coordination of the action within a person) and interpersonal coordination (the coordination of the action with another person) (Kovacs et al., 2020) in time and space is necessary for such joint actions to be successful (Sebanz et al., 2006). Many sub-actions are performed during handover actions including both feedforward and feedback control mechanisms to use predictions to anticipate one’s motor executions, as well as to implement error corrections. A detailed understanding of the motor control processes of both the giver and receiver that underlie handover actions and the factors that influence them contribute to the testing of concepts of human interaction and further development of robotic technologies. Thus, the article aims was to provide a common framework for investigating handover actions, based on an overview of the current state of research on handover actions. To facilitate this, we first divided handover actions into discrete phases and named them to create a foundation for clear communication.

Joint actions are an essential part of human life and are characterized by the fact that two or more individuals pursue a common goal and coordinate their individual actions accordingly. This coordination requires an optimal alignment of the actors in time and space. To achieve this, additional abilities beyond those required in a single action are necessary. These abilities are (a) the sharing of representations, (b) the prediction of actions of the co-actor, and (c) the continuous integration of predictions and incoming information (Sebanz et al., 2006). Shared representations, common mapping of external conditions (Hagendorf et al., 2011), are formed through the planning of one’s own actions and predicting that of one’s partner (Kourtis et al., 2014), while considering the constraints of both (Schmitz et al., 2017). The individual constraints of one’s own body and that of one’s partner, such as body size or obstacles in the action space, are considered during this process. Based on these shared representations, predictions about the co-actor’s actions are made that are then used for anticipatory action control. The predictions are integrated into the available perceptual information (i.e., feedback control), enabling coordination in time and space (Sebanz and Knoblich, 2021). In this context, incoming information means monitoring one’s own actions and the actions of one’s partner to identify discrepancies between the expected and actual execution (Loehr et al., 2013). For example, when taking the role of the receiver in a handover task, misjudgments about the anticipated movement trajectories of the giver are detected through constant observation and by monitoring the giver’s movement kinematics, thus the response plan may be updated accordingly. In the same vein, the receiver may anticipate the essential properties of the handover object (such as its weight). Information and even misjudgments about these properties (e.g., an empty milk carton, instead of the expected full carton) may also be detected in the action-partner’s movement kinematics with, for example, heavy objects leading to different kinematics than light objects (Eastough and Edwards, 2007). Thus, the receiver may be able to develop an accurate forward model that enables the precise anticipatory scaling of grip forces needed to successfully grasp the object.

Movement kinematics (which can be measured with 3D motion tracking systems) such as movement duration (Vesper et al., 2017), height, and velocity (McEllin et al., 2018) contain relevant information for the receiver of an object in a handover task. This means that an actor transmits information through the way they move during an action. This can be viewed as signaling (i.e., an intentional communication strategy) through which the actor makes their task execution more predictable for the co-actor in order to minimize uncertainties in the prediction of their action and, thus, optimize the joint action (Pezzulo and Dindo, 2011). The information required for the joint action can be communicated, for example, by varying the motor executions and systematically deviating from the most efficient way of executing the action (Pezzulo et al., 2013) (e.g., by changing in the duration or velocity of a certain action). Such signals could be used in a handover action, for example, to communicate the position of the handover. In addition to signaling for action synchronization, other environmental factors can also lead to an observable change in kinematics. Assuming that the reaching and grasping of the giver are influenced by specific factors (e.g., object properties) (Yamamoto et al., 2016), the receiver may obtain information about these factors by observing such movements (Lastrico et al., 2021). Observing how a person grasps an object and transports it to the handover position can provide information about the weight or fragility of an object and even the handover position, whereupon the receiver can perform a more precisely adapted action (e.g., more precise initial grip force scaling).

Research on joint handover actions was not only of interest to psychologists and movement scientists, it is also pose a major challenge in robotics research today (Thomaz et al., 2016; Castro et al., 2021). Thus, investigating human handover actions may help determine key features of the kinematics of human handover movements (Liu et al., 2021) and, thus, enable the robot to interpret human behavior and adapt its own movement to human requirements. Furthermore, the results of the investigations of human-human handover actions can be used to design robots in such a way that they act more human-like so that the human-robot interaction will be perceived as more natural from the human perspective (Costanzo et al., 2021). As empirical experiments on handover actions are being conducted in robotics, movement science, and psychology, we are proposing a common terminology and framework that will facilitate scientific exchange and, thus, advance research in this area. Language is a key challenge in the context of interdisciplinary works, thus, it is advisable to create a clear framework description and, thus, a common language (Wear, 1999; Domino et al., 2007). To the best of our knowledge, no common framework has yet been established for research on handover actions, with the result that various terms are being used to describe one meaning, while different meanings are being attributed to other specific terms.

The aim of this review was, therefore, to provide a foundation for interdisciplinary research in the field of handover actions. To this end, the current literature on handover actions was systematically reviewed and a common framework was derived that clearly defines the individual sub-actions of a handover action, thus faciliating the clear identification of the different components of handover actions. The systematic literature review also provided an overview of characteristics in the execution of human-human handover actions and enabled us to identify different factors, such as object properties, that influence the execution of a handover action and to identify in what fashion they influence the action.

## 2. Methods

### 2.1. Transparency and openness

This study followed the Transparency and Openness Promotion (TOP) Guidelines (Level 2; Mellor et al., 2022). The systematic review was also performed according to PRISMA guidelines (Moher et al., 2009). All references have been cited according to the maximum level of uniqueness (if a DOI was available, this has been included). No original data has been used, hence, there are no ethical constraints on data sharing.

### 2.2. Search strategy

The literature search was conducted in June 2021, with the final update on June 16, 2021. Based on a preliminary search of relevant publications in the field of handover action, we decided to include items published between January 1980 and June 16, 2021, in German and English from the databases PsychINFO, PubMed, Scopus, and Web of Science. To optimally adapt the search term formula to the research question, the individual search terms were combined with the operators “AND” or “OR.” The search was carried out within titles, abstracts, and keywords. The search term formula used was: (hand-over OR handover* OR pass OR passing OR transfer* OR “joint*”) AND (object OR objects) AND (kinematic OR kinematics OR force OR forces OR “motion*” OR “grasp*” OR “grip*” OR social) AND (“human*” OR “participant*”).

### 2.3. Selection criterion

For the purpose of our systematic literature review, we considered studies that empirically investigated handover actions between two human actors. A handover action was considered as such if both actors had an active part (i.e., giver reduced grip force, receiver increased grip force) during the object transfer phase (the part of the handover action in which both actors had physical contact with the object). As we were referring to the execution of a handover action, we included studies that recorded at least one of the two data types kinematics or grip force of one or both of the actors. Dissertations, conference papers, case studies that were not peer-reviewed, and studies that did not produce an outcome of interest were excluded from this review.

### 2.4. Selection process and data extraction

First, all duplicates were removed from the set of publications gathered using the search term formula above and the title and abstracts were scanned. Potential publications were then screened by two independent researchers in relation to the predefined inclusion and exclusion criteria. The remaining studies were assessed for their eligibility and when disagreements occurred between the two researchers, a third, independent researcher was consulted.

### 2.5. Definition of a handover action

Given that studies focused on a variety of different objectives in handover actions, they used diverse experimental setups and procedures. As some studies claimed to have investigated handover actions, but the experimental design did not exhibit an actual handover action (e.g., an object was replaced by one subject followed by another subject grasping the object), we include or exclude studies based on the following definition:

The handover action should comprise a transfer phase in which both actors (giver and receiver) have physical contact with the object at the same time. Furthermore, both actors must have an active part in the transfer phase. Hence, it is not sufficient if only one actor is active (e.g., one person takes/pulls an object out of another person's hand).

### 2.6. Assessment of methodological quality

Following the recommendation that Ma et al. (2020) make regarding cross-sectional studies, a quality assessment was performed using the Joanna Briggs Institute tool (Moola et al., 2017). The criteria considered were (a) subject selection, (b) the description of subjects and, setting, (c) validity/reliability, (d) the objectivity of measurement, (e) control of confounding factors, (f) validity/reliability of outcomes, and (g) the appropriateness of statistics used. The results of the quality assessment of each study are summarized in Table 1.

**Table 1:** Study characteristics.

First author	Sample	Study design	Aim	Conditions	Type of data	Measuring instruments	Handover object	Results	Risk of bias
Becchio et al. (2008)	*N* = 13 f/m = 11/2 age = 20–31 h = r	Italy Giver *n* = 30 Within-subjects study design	Influence of intention on the execution of action	3 tasks (single action, social, passive-observer)	Kinematics (wrist, index, thumb)	ELITE – Bioengineering technology and Systems (4 cameras)	Egg-shaped object	Longer duration of finger closure when grasping in social than in single action condition Higher point of maximum trajectory height and shorter time to maximum velocity in social than in single action condition	Moderate
Bekemeier et al. (2019)	*N* = 10 f/m = 5/5 age = 24–73 h = n.a.	Germany Giver/receiver *n* = 96 Within-subjects study design	Classification of identity and personality characteristics by handover trajectories	2 handover heights (low, high)2 object sizes (low, high)2 object weights (light, heavy)2 types of handover (direct, indirect)2 roles (giver, receiver)	Kinematics (hand)	Vicon (17 cameras)	Beaker (differs in size and weight)	Classification of identity possible Classification of personality characteristics not possible	Low
Cini et al. (2019)	*N* = 34 f/m = 11/23 age = 30.9 (7.9) h = r	Australia Giver/receiver *n* = 306 Within-subjects study design	Choice of grasp type and hand placement on object during handover	2 activities (non-interactive, interactive) 2 tasks (replacement, use)	Kinematics (grasp classification)	Optitrack (10 cameras)	17 Everyday objects	Precision grip was chosen in 73.6% of interactive trials and 50.9% of non-interactive trialsGiver and receiver choose similar grip types but receivers more frequently use power grip than giver	Moderate
Controzzi et al. (2018)	*N* = 14 f/m = 5/9 age = 26 (11) h = r	Italy Giver *n* = 60 Within-subjects study design	Investigates the need for the givers’ visual input on anticipatory control to trigger the release of the object	3 receiver’s reaching velocities (slow, medium, fast)2 giver’s visions (available, not available)	Grip force	2 Six-axis force/torque sensors	Abstract object	Giver starts releasing in synchrony with object-receiver contact Grip force releasing rate correlates with receivers reaching velocity Without vision: Start of grip force releases delayed proportionally to the receivers reaching velocity	Low
Döhring et al. (2020)	*N* = 22 f/m = 16/6 age = 23.4 (2.4) h = r	Germany Giver *n* = 128 Within-subjects study design	Influence of available sensory information on grip force control	4 receiver’s reaching velocities (slow, medium, fast, very fast)2 giver’s visions (available, not available)2 giver’s tactile information (glove, no glove)	Grip force	2 Strain-gauge-sensors	Abstract object	Rate of grip force release increases with reduction of sensory information (most due to removal of tactile information) Handover duration increases with reduction of sensory information (mostly due to removal of visual information) Receiver grip force rate proportional to reaching velocity Giver adapts their force rates to receivers’ force rates	Low
Gonzalez et al. (2011)	*N* = 10 f/m = 1/9 age = 32.2 (11.1) h = 8 r, 2 l	USA Giver *n* = 216 Within-subjects study design	Influence of the partner’s intention on one’s own motor planning	3 objects (hammer, calculator, stick)2 tasks (non-interactive, interactive)2 initial orientations (comfortable, uncomfortable)2 tasks (replacement, use)	Kinematics (hand placement on object)	Panasonic MiniDV camera (video camera)	Toy hammer Calculator Stick	Maximization of comfort in own end-state and beginning state of the partner	High
Hansen et al. (2017)	*N* = 10 f/m = 4/6 age = 26.0 (5.0) h = r	France Giver/receiver *n* = 18 Within-subjects study design	Influence of object weight and interactor distance on handover kinematics	3 object weights (light, medium, heavy)3 inter-actor distances (self-chosen, self-chosen +20%, self-chosen - 20%)2 roles (giver, receiver)	Kinematics (hand)	Vicon (17 cameras)	Dry food jars	Distance and mass affect handover duration Distance affects handover height Mass does not affect handover height	Low
Mason and Mackenzie (2005)	*N* = 12 f/m = 6/6 age = 18–23 h = r	Canada Giver/receiver *n* = 80 Within-subjects study design	Initial grip force scaling of giver and receiver Mutual influence of the kinematics of the actors	2 giver’s reaching behaviors (stationary, moving) 2 receiver’s reaching behaviors (stationary, moving) 2 roles (giver, receiver)	Grip force Kinematics (hand, wrist)	2 Load CellsOptotrak (2 cameras)	Rectangular object	Giver kinematics influences receivers’ kinematics Receiver kinematics do not influence giver kinematics Synchronization of grip forces in object transfer phase	Low
Meyer et al. (2013)	*N* = 44 f/m = 34/10 age = n.a. h = n.a.	Netherlands Giver *n* = 36 Within-/between-subjects study design (individual-joint group, joint-joint group)	Is the end-state comfort of the receiver considered by the giver?	3 object end positions (low, medium, high) 6 object types (differently arranged grasp areas)	Kinematics (grasping area on object: low vs. high)	Video camera	Cylinders with different grasping areas	Givers consider the end-state comfort of the receiver Learning effect in third-order planning that can be transferred from individual (own experience with end-state comfort) to joint actions	Low
Sutiphotinun et al. (2020)	*N* = 20 f/m = n.a. age = n.a. h = r	Thailand Giver/receiver *n* = 15 subjects are standing/walking Within-subjects study design	How do the giver and receiver find the handover position, during a handover action? What strategies do agents use during the transfer phase under varying object weights? How does the giver regulate the bilateral force before releasing the object?	3 object weights (light, medium, heavy)	Grip force Kinematics (object)	Multi-axis force sensor	Bottle	Handover actions consist of three distinct phases (send, transfer, receive) Giver kinematics in agreement with minimum jerk theory (unaffected by object weight) Body size influences the handover position	High
*Endo et al. (2012)	*N* = 10 f/m = 5/5 age = 31.4 (5.6) h = r	United Kingdom Giver/receiver *n* = 140 Within-subjects study design	Effect of uncertainty about partner’s movement on grip forces	3 handover locations (middle, close to giver, close to receiver)3 orders of handover locations (natural, fixed, random)3 receiver’s tactile information (glove/no glove)	Grip force Kinematics (wrist)	3 6-DoF force/torque sensor Qualisys (12 cameras)	Abstract object	Givers start the grip release later when the handover position varies randomly or the receiver wears a glove Forces at contact dropped across trials	Moderate

N: Number of participants; f/m: Number of female/male; age: Participants’ age in years; h: handedness (l: left; r: right); n: Number of trials per participant *: Supplementary study that does not fulfill the inclusion criteria but is relevant in terms of content.

## 3. Results

### 3.1. Search results

As a result of our electronic database search, in PsychINFO, PubMed, Scopus, and Web of Science, a total of 9,092 studies were identified. All studies were found, which we had also previously found in our preliminary search.

**Figure 1:**
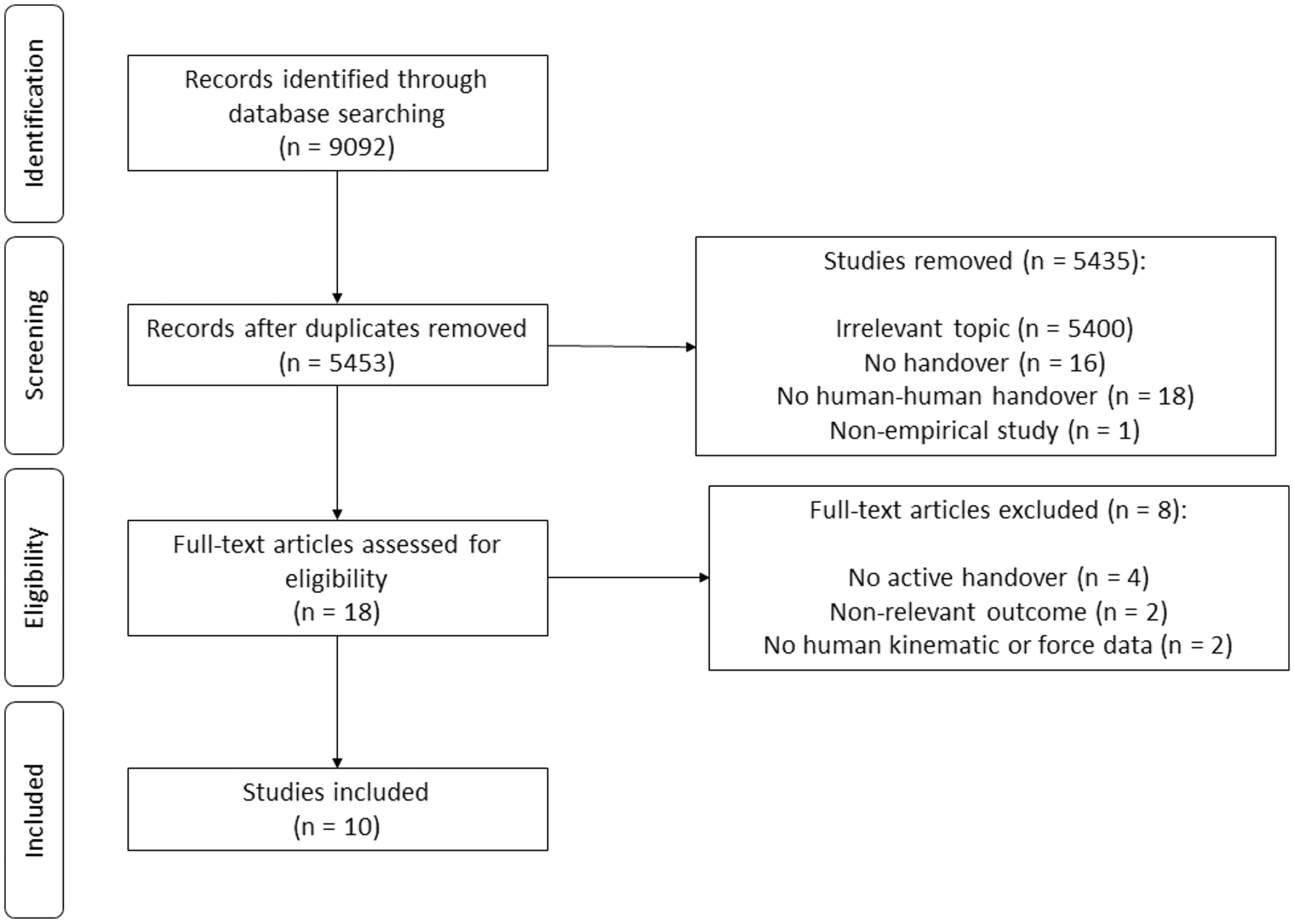
PRISMA flow chart of the research process.

After removing duplicates (*n* = 3,639 removed) and after title and abstract screening (*n* = 5,435 removed), the full text of 18 studies were scanned and 10 studies were found to meet our eligibility criteria [*n* = 8 removed: no active handover = 4 (Salleh et al., 2011; Parastegari et al., 2018; Kato et al., 2019; Neranon, 2020), non-relevant outcome = 2 (Korkiakangas et al., 2014; Carfì et al., 2019), no human kinematic or force data = 2 (Xie and Zhao, 2015; Chan et al., 2020)]. Thus, we included 10 studies in our systematic review that investigated the characteristics of human-human handover actions and their influencing factors, such as the handover object weight and availability of sensory information (Mason and Mackenzie, 2005; Becchio et al., 2008; Gonzalez et al., 2011; Meyer et al., 2013; Hansen et al., 2017; Controzzi et al., 2018; Bekemeier et al., 2019; Cini et al., 2019; Döhring et al., 2020; Sutiphotinun et al., 2020). See Figure 1 for a comprehensive flowchart of our search process. In the following section, we detail studies with regard to specific study characteristics, such as study design and participant characteristics (e.g., age, gender), experimental task and condition/manipulation, and outcome parameters of interest.

In scanning the references of the included articles, a conference paper was found that was relevant to the context of this review (Endo et al., 2012). As conference papers were excluded, this paper was not considered in the results section. Nevertheless, it is a detailed conference paper that contained a comprehensive description of the study methodology and has, therefore, been included in the discussion of the complete overview of handover research and added to Table 1.

### 3.2. Study design and participant characteristics

The main study characteristics identified in our sample are summarized in Table 1. The selection of studies showed strong variations in the scope of their design and research aims. Therefore, we included additional information on the aims of each study and the handover object in the table, supplementing that information as recommended in the PRISMA guidelines (Moher et al., 2009). All the studies included were published between 2005 and 2021 and were conducted in eight different countries (Germany, Italy = 2, Australia, Canada, France, Netherlands, Thailand, USA = 1 each). Study designs varied, particularly, in the role that was allocated to the subjects. In five studies, the subjects took the role of both, giver and receiver. In three of these studies, subjects switched the giver/receiver roles in the course of the experiment (each subject was giver and receiver in 50% of the trials; Mason and Mackenzie, 2005; Hansen et al., 2017; Bekemeier et al., 2019). In the other two studies, the roles were permanently assigned (Cini et al., 2019; Sutiphotinun et al., 2020). In the remaining five studies, the experimenter took the role of the receiver and the subjects were only assigned the role of the giver (Becchio et al., 2008; Gonzalez et al., 2011; Meyer et al., 2013; Controzzi et al., 2018; Döhring et al., 2020). All included studies were conducted using a within-subjects design (Mason and Mackenzie, 2005; Becchio et al., 2008; Gonzalez et al., 2011; Meyer et al., 2013; Hansen et al., 2017; Controzzi et al., 2018; Bekemeier et al., 2019; Cini et al., 2019; Döhring et al., 2020; Sutiphotinun et al., 2020), although Meyer et al. (2013) also used a between-subjects design. In this study, Meyer and colleagues divided their subjects into two different groups, who were the assigned different tasks in the first part of the study. Half of the subjects initially had a single action task (replacement) and then a joint action task (handover), while the other half had to complete a joint action task (handover) in both parts of the investigation.

Overall, data was collected from 189 individuals with sample sizes per study ranging from 10 to 44. In one study with 20 participants, no gender distribution was given (Sutiphotinun et al., 2020), so these study participants are not included in the gender description of the sample. Across the remaining studies, the gender distribution was relatively balanced with 55% female and 45% male participants. The age reported in the individual studies ranged from 18 to 32 years (Mason and Mackenzie, 2005; Becchio et al., 2008; Gonzalez et al., 2011; Hansen et al., 2017; Controzzi et al., 2018; Bekemeier et al., 2019; Cini et al., 2019; Döhring et al., 2020), with the exception of one study that also included two subjects over 70-years-old and one subject over 40-years-old (Bekemeier et al., 2019). As the age of the subjects was reported in different ways, it is not possible to determine a mean value across all studies. Two studies did not specify the age of their subjects (Meyer et al., 2013; Sutiphotinun et al., 2020). In most studies, all the subjects were right-handed (Mason and Mackenzie, 2005; Becchio et al., 2008; Hansen et al., 2017; Controzzi et al., 2018; Cini et al., 2019; Döhring et al., 2020; Sutiphotinun et al., 2020), and only one study also included two left-handed subjects (Gonzalez et al., 2011). Two studies did not report any information about the handedness of the subjects (Meyer et al., 2013; Bekemeier et al., 2019).

### 3.3. Research areas and terminology

The diversity of disciplines interested in handover actions mapped out in our introduction is reflected in the disciplinary background of the studies considered in this review. The studies were conducted by scientists from the fields of movement science (Mason and Mackenzie, 2005; Gonzalez et al., 2011; Hansen et al., 2017; Döhring et al., 2020), psychology (Becchio et al., 2008; Meyer et al., 2013; Controzzi et al., 2018), informatics (Bekemeier et al., 2019), and robotics (Hansen et al., 2017; Controzzi et al., 2018; Cini et al., 2019; Sutiphotinun et al., 2020).

Consequently, the terminology used in the studies is rather inconsistent. The term “handover” as defined in this review was used in the same way in five studies (Hansen et al., 2017; Bekemeier et al., 2019; Cini et al., 2019; Döhring et al., 2020; Sutiphotinun et al., 2020), while the other studies used the terms “object passing” (Mason and Mackenzie, 2005; Becchio et al., 2008; Gonzalez et al., 2011; Controzzi et al., 2018) or “joint object manipulation” (Meyer et al., 2013) synonymously.

In addition, there were variations in how studies referred to the two actors. In three studies, no names were assigned at all to either actor (Becchio et al., 2008; Gonzalez et al., 2011; Meyer et al., 2013). However, in all other studies, the word “receiver” was used uniformly for the person receiving the object (Mason and Mackenzie, 2005; Hansen et al., 2017; Controzzi et al., 2018; Bekemeier et al., 2019; Cini et al., 2019; Döhring et al., 2020; Sutiphotinun et al., 2020), while either the term “giver”, (Hansen et al., 2017; Bekemeier et al., 2019; Sutiphotinun et al., 2020) or “passer” (Mason and Mackenzie, 2005; Controzzi et al., 2018; Cini et al., 2019; Döhring et al., 2020) was used to refer to the person giving the object.

The most important inconsistency across the studies was, however, the division of a handover action into specific phases from grasping the object to having completed the handover. Three studies did not divide the action into phases (Gonzalez et al., 2011; Meyer et al., 2013; Bekemeier et al., 2019). The remaining studies differed both in terms of the number of phases (between two and five) and in the temporal events demarking the onset and termination of the individual phases. Cini and colleagues (Cini et al., 2019) divided handover actions into two phases, (1) the “handover” and (2) the “subsequent action”, where the handover phase ends with the giver losing contact with the object (and the object remaining in the receiver’s hand; Cini et al., 2019). In contrast, Becchio and colleagues (Becchio et al., 2008) called the phases (1) “reach-to-grasp” and (2) “place”. The “reach-to-grasp” phase describes the part until the giver has grasped the object and the object starts to move. At this point, the “place” phase begins. Mason and Mackenzie (2005) also divided the action into two phases called (1) “object transport by passer/reach to grasp by receiver” and (2) “object transfer”. The first phase ends with the first contact between the receiver and the object. This point also marks the beginning of the second phase, which ends as soon as the giver loses contact with the object (Mason and Mackenzie, 2005). Controzzi et al. (2018), Döhring et al. (2020), and Sutiphotinun et al. (2020) presented a division into three phases. Similar to Mason and Mackenzie’s (2005) division, the first phase ends with the first contact between the receiver and the handover object. However, they each had a different term for it, ranging from “coordination” (Controzzi et al., 2018), “transport phase passer” (Döhring et al., 2020), to “sending” (Sutiphotinun et al., 2020). The second phase describes the time in a handover action in which both actors have physical contact with the object. It begins with the end of the first phase and ends when the giver loses contact with the object (similar to the object transfer phase of Mason and Mackenzie (2005)). This phase was called “modulation of grip forces” (Controzzi et al., 2018), “handover” (Döhring et al., 2020), or “transferring” (Sutiphotinun et al., 2020). Similar to the subsequent action phase of Cini et al. (2019), the third and final phase of the handover action describes the phase where the object remains in the receiver’s hand. This is called “end of handover” (Controzzi et al., 2018), “transport phase receiver” (Döhring et al., 2020), or “receiving” (Sutiphotinun et al., 2020). Hansen et al. (2017) divided the handover action into five phases. Their phase divisions only consider the actions of the giver and are called (1) “reaching”, (2) “loading”, (3) “in-hand manipulation” (comparable to the first phase according to Mason and Mackenzie (2005), Controzzi et al. (2018), Döhring et al. (2020), and Sutiphotinun et al. (2020), (4) “release”, and (5) “unloading”. The beginning and end of the phases are not described in more detail (Hansen et al., 2017). This variation in how the handover movement has been divided into phases makes comparability across studies arduous.

### 3.4. Experimental task and condition/manipulation

Given the diverse objectives of the individual studies, they also varied with respect to the type of data collected and the manipulation of the experimental conditions. Seven studies recorded kinematic data, using different measurement techniques such as 3D motion tracking (Mason and Mackenzie, 2005; Becchio et al., 2008; Hansen et al., 2017; Bekemeier et al., 2019; Cini et al., 2019) and video cameras (Gonzalez et al., 2011; Meyer et al., 2013; Sutiphotinun et al., 2020). Four studies recorded the grip forces exerted on the handover object (Mason and Mackenzie, 2005; Controzzi et al., 2018; Döhring et al., 2020; Sutiphotinun et al., 2020), while two studies assessed both kinematic and dynamic data (Mason and Mackenzie, 2005; Sutiphotinun et al., 2020).

In addition to the handover action, an additional comparison task was performed in four studies (Becchio et al., 2008; Gonzalez et al., 2011; Meyer et al., 2013; Cini et al., 2019). In one study, a replacement task (single action condition) was compared with a similar handover task (social condition; Becchio et al., 2008). In the other three studies, the comparison task was a single action task with two different conditions, namely (1) replacement or (2) use (Gonzalez et al., 2011; Meyer et al., 2013; Cini et al., 2019). This extension enabled a comparison between single and joint actions. In one study, the control task was investigated using a between-subjects design (see Section “Study design and participant characteristics”; Meyer et al., 2013). In the other three studies, the control task was investigated within subjects, for givers only (Becchio et al., 2008; Gonzalez et al., 2011; Cini et al., 2019). In a number of studies, the object properties were systematically varied. This included the size of the object (Bekemeier et al., 2019), the weight of the object (Hansen et al., 2017; Bekemeier et al., 2019; Sutiphotinun et al., 2020), or the type of object, i.e., different everyday objects were used (Gonzalez et al., 2011; Meyer et al., 2013). In addition, one study manipulated the starting position of the object (comfortable vs. uncomfortable; Gonzalez et al., 2011) and another study varied the final position of the object, i.e., a low, medium, or high shelf (Meyer et al., 2013).

The handover position, i.e., the position of the object during the phase in which both subjects had physical contact with the object, was also systematically manipulated in two studies. These manipulations included the height of the handover position (Bekemeier et al., 2019) and the distance between the actors (Hansen et al., 2017). The height was manipulated using a wooden obstacle that forced participants to perform the handover task at a higher position than without the obstacle.

Other studies manipulated the behavior of the actors. In two studies, in which the experimenter took over the role of the receiver, the reaching velocity to the handover position was varied (Controzzi et al., 2018; Döhring et al., 2020). In another study, researcher demonstrated the influence of the behavior of both receivers and givers by asking subjects to either remain stationary or move during the handover (Mason and Mackenzie, 2005). When remaining stationary, the subject placed their hand in the handover area at the start of the trial. When moving, the subject’s hand was placed in a starting position close to the subject’s body at the beginning of the trial.

Manipulation of sensory input was used on both the giver and the receiver in four studies. Two studies manipulated the giver’s visual input through blindfolding (Controzzi et al., 2018; Döhring et al., 2020), while another study manipulated the giver’s haptic input (Döhring et al., 2020) using gloves.

Overall, the experimental set-up varies significantly between the studies. Depending on the research question the individual studies sought to address, two different types of data were recorded (kinematics and/or grip forces), the focus was either on both actors or only one actor (giver or receiver), and different elements of the handover action were manipulated including object properties, distance between the actors, behavior of the co-actor, etc.

### 3.5. Outcome parameters of kinematics

Eight studies recorded kinematic data, using different measurement techniques, such as 3D motion tracking (Mason and Mackenzie, 2005; Becchio et al., 2008; Hansen et al., 2017; Bekemeier et al., 2019; Cini et al., 2019) and video cameras (Gonzalez et al., 2011; Meyer et al., 2013; Sutiphotinun et al., 2020).

The study by Becchio et al. (2008) shows differences between a single and a joint action. Already while the giver is grasping the object, there is a difference between the two tasks. In the joint task, the giver needs more time to enclose the object with the fingers than in the single task. While the giver is transporting the object to the handover position, the maximum height of the object is higher, as well as the time to reach the maximum velocity is shorter in the joint task. This indicates that accurate placement of the fingers on the object and more accurate trajectory is necessary to ensure optimal handover. This is also consistent with the result of Cini and colleagues. According to the study by Cini et al. (2019), givers were more likely to use a precision grip in a handover action than in single action tasks (e.g., the replacement task). In addition, when objects had a handle, it was left free for the receiver when possible (Cini et al., 2019). Furthermore, the analyses of grasping patterns in three studies (Gonzalez et al., 2011; Meyer et al., 2013; Cini et al., 2019) suggested that givers consider the receiver’s beginning and end-state comfort (not exclusively their own). This means that if the subsequent activity intended by the receiver was known by the giver, they took this into account in their own grasping behavior so that the receiver was able to perform their subsequent activity in a comfortable manner. Contrary to the giver, there was no discernable difference in the receiver’s grasp in comparison to a single action task (Cini et al., 2019).

Bekemeier et al. (2019), also analyzed the movement kinematics and revealed that the trajectories in handover actions exhibited a high degree of individuality. Thus, it was possible to identify a participant by observing the movement trajectories. Furthermore, intrapersonal variations in kinematics (i.e., changes in kinematics within a person) were observed when the object properties or the role (giver vs. receiver) were manipulated. Although the variance in the trajectories increased when object properties were manipulated, the subjects could still be classified based on the individuality of their movements. This increased variation was mostly caused by the object weight, i.e., the heavier the object, the larger the variation in the trajectories. Furthermore, analysis of the trajectories could also be used to identify the classification of the experimental manipulations (Bekemeier et al., 2019).

Two other studies tested the influence of object weight on kinematics (Hansen et al., 2017; Sutiphotinun et al., 2020). Both studies investigated whether the handover position was influenced by the object weight and one of the two studies investigated whether the velocity profiles were influenced by the object weight (Sutiphotinun et al., 2020). The velocity profile (Sutiphotinun et al., 2020) and handover position were not affected by the object weight. However, it was shown that the handover took place in a horizontal plane at the center of the actors (both anterior–posterior and medio-lateral) (Hansen et al., 2017). The height of the handover position was influenced by the distance between the two actors (the further away they were, the lower the handover height) (Hansen et al., 2017) and the height of the actors (the taller the actors, the higher the handover height; Sutiphotinun et al., 2020), but not by the object’s weight (Sutiphotinun et al., 2020). However, while the object weight did not influence the handover position, it did in fact influence the duration of the transfer phase, with greater object mass yielding longer transfer (Hansen et al., 2017).

Regarding the influence of kinematics on handover actions, it can be concluded that the intention (i.e., why or for what purpose the object is handed over; Gonzalez et al., 2011; Meyer et al., 2013; Cini et al., 2019) and individuality (Bekemeier et al., 2019; Sutiphotinun et al., 2020) of the actors influenced the kinematics of a handover action. In contrast, the influence of object weight on the kinematics seemed to be ambiguous. While it has been shown that the hand trajectory of the giver as they moved the object to the handover position changed systematically in relation to object weight (became more variable and took longer; Bekemeier et al., 2019), other studies have shown that neither the velocity profile (which was contained in the trajectory) nor the handover position (which was also contained in the trajectory through spatial data) were influenced by object weight (Hansen et al., 2017; Sutiphotinun et al., 2020).

### 3.6. Outcome parameters of dynamics

Only four studies recorded the grip forces exerted on the handover object (Mason and Mackenzie, 2005; Controzzi et al., 2018; Döhring et al., 2020; Sutiphotinun et al., 2020). Therefore, they focused on the object transfer phase, meaning the part of the handover action where both the giver and the receiver have physical contact with the handover object. It was shown that the grip forces of the giver and receiver synchronized in such a way that the rate of change in grip force was similar in the giver (reduction of grip force) and receiver (increase of grip force; Mason and Mackenzie, 2005; Controzzi et al., 2018).

The duration of the transfer phase was not only affected by the object weight (see Section “Outcome parameters of kinematics”), but also by the availability of visual information from the giver (Controzzi et al., 2018; Döhring et al., 2020). The removal of visual information led to a delay in the giver’s grip force reduction, which resulted in a longer transfer time. In contrast, if the haptic input was reduced through the use of a glove, the transfer duration or the giver’s grip force reduction was not affected (Controzzi et al., 2018).

The receiver’s reach-to-grasp velocity (prior to the actual object transfer) affected the duration of the transfer as well. The faster the receiver moved their hand to the handover position, the greater the giver’s grip force release rate was (Controzzi et al., 2018; Döhring et al., 2020). The synchronization of the grip forces was maintained, even when there were variations in the receiver’s reaching behavior (Mason and Mackenzie, 2005; Controzzi et al., 2018; Döhring et al., 2020).

### 3.7. Methodological quality

In relation to the risk of bias assessment, six studies were classified as having a low risk of bias (Mason and Mackenzie, 2005; Meyer et al., 2013; Hansen et al., 2017; Controzzi et al., 2018; Bekemeier et al., 2019; Döhring et al., 2020), two as having a medium risk of bias (Becchio et al., 2008; Cini et al., 2019), and two as having a high risk of bias (Gonzalez et al., 2011; Sutiphotinun et al., 2020). In most cases, the risk of bias was introduced by not reporting confounding factors and considering how to deal with them.

## 4. Discussion

In this systematic review, provided an overview of studies on handover actions and the characteristics derived from them. In total, ten studies were found in which experiments were conducted on handover actions between two human actors. Overall, only a small number of human-human handover experiments have been conducted to date that have sought to answer a broad spectrum of different research questions. Accordingly, the methodology used to conduct the experiments also differed significantly. Therefore, in order to create a unifying language that will serve as a conceptual basis for a synthesis of the results (as well as for future studies), a common framework for handover actions is provided in the first part of this discussion section. This framework is then subsequently used to interpret and discuss the results of the synthesized studies with respect to the individual distinct phases of a handover action.

### 4.1. Creating a common framework

Handover actions, as an experimental paradigm, have been researched in a range of different scientific disciplines (movement science, psychology, informatics, and robotics). Consequently, the theoretical embedding and research aims of the studies on human handovers vary greatly and no uniform terminology has emerged - until now. Therefore, we present a common framework that has been derived from the questions and results of the studies that were presented in the results section. The intention is to make the description in future studies simpler, shorter, and more precise.

A handover action is performed by two persons acting together. At the beginning of the handover action, the first acting person moves their hand toward the object: This person is called the giver. The person who accepts the object to be transferred from the giver is called the receiver.

**Figure 2:**
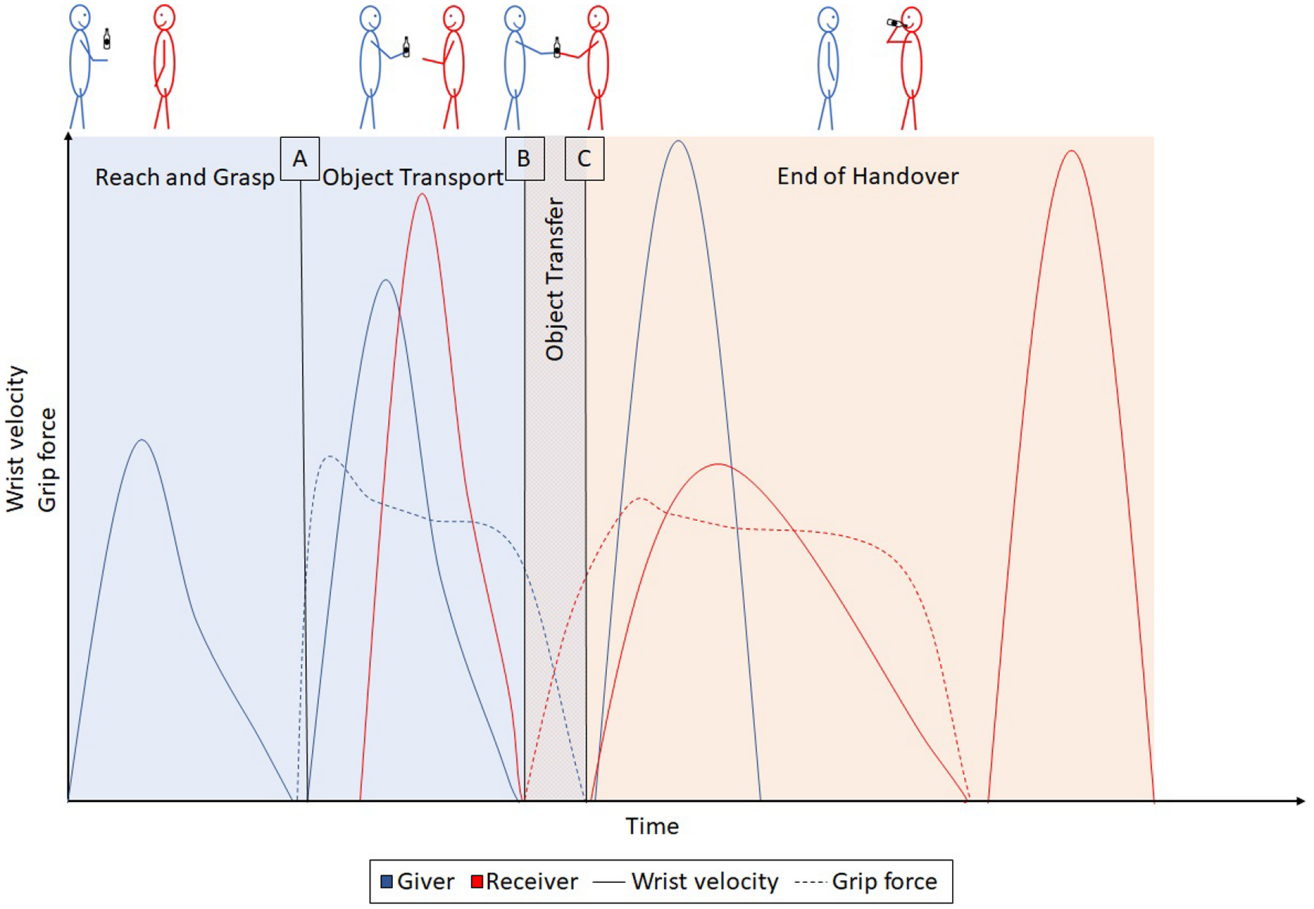
Exemplary, symbolic illustration of the kinematics (solid) and dynamics (dashed) of the giver (blue) and receiver (red) in a handover action based on information from Controzzi et al. (2018), Döhring et al. (2020), Endo et al. (2012), and Mason and Mackenzie (2005). **(A)**, marks the end of the reach and grasp phase and the beginning of the object transport phase. **(B)**, marks the end of the object transport phase and the beginning of the object transfer phase. **(C)**, marks the end of the handover.

The actors perform successive actions, however, the actions of each actor partially overlap in time (see Figure 2). Based on distinct temporal events within handover actions, they can be divided into clearly distinguishable phases. To achieve this, we have considered the different phase divisions of the studies described thus far, brought them together, and attempted to separate them unambiguously into the specific events within a handover action. The first phase of a handover action is the “reach and grasp phase.” In this phase, the giver reaches out to the object, grasps it, and increases the grip force until the required force is reached. The reach and grasp phase ends when the necessary grip force is reached, that is immediately before the object is moved and loses contact with the ground. This is followed by the second “object transport phase” in which the giver moves the object from its starting place to the handover position. Typically, the receiver starts their action during the object transport phase when they reach toward the handover position. The object transport phase ends as soon as the receiver makes physical contact with the object. This marks the beginning of the third “object transfer phase”, which is the core phase of the handover action. In this phase, the receiver builds up grip force until they alone hold the object in their hand, while the giver simultaneously reduces their grip force until they lose contact with the object. As soon as the giver loses physical contact with the object, the object transfer phase is finished. The object transfer phase is the end of the actual handover action. However, another subsequent, fourth phase is described in this proposed framework, the “end of handover” phase. The actions at the end of the handover phase take place after the handover action is complete but, nevertheless, influence the previous phases. Thus, whether or not an object will be used by the receiver after a handover action can be used as a manipulation for an experimental setup. As this phase influences the previous actions, it is advisable to consider it in the common framework. In this phase, the giver returns their hand to the rest position and the receiver executes the intended action (e.g., repositioning or tool use).

### 4.2. The reach and grasp phase

At first glance, the reach and grasp phase of a handover action does not appear to differ significantly from the reach and grasp phase of a single action (e.g., an object manipulation action). During reach and grasp., both the hand is moved toward the object and simultaneously the hand is opened to grasp until the fingers wrap around the object (Jeannerod, 1981, 1984). Different grip patterns are possible, here we only distinguish between the two categories “precision grip” and “power grip” (Napier, 1956). The precision grip is characterized by the fact that the thumb and fingertips oppose each other. In the power grip, the object is held between the thumb, finger and palm; direct contact of the fingertips with the object is not necessary. Both the choice of the grasp pattern and grasp location are greatly influenced by the object’s properties. The object’s size, shape, weight, and orientation all play an important role (Napier, 1956; Feix et al., 2014). However, another factor that influences the choice of grasp type and location is the intention with which the object is being grasped (Napier, 1956).

When comparing single and joint actions with the same objects, it was shown that individuals act more cautiously (Becchio et al., 2008) and tended to choose a precision grip rather than a power grip when they wanted to hand over an object (Cini et al., 2019). A precision grip may have several advantages over a power grip. First, the fingertips (mainly involved in precision grip, less involved in power grip) represent the areas of the hand that have the highest density of mechanoreceptors (Johansson and Vallbo, 1983; Vallbo and Johansson, 1984). This means that by choosing a precision grip, there is a higher sensitivity to the applied forces resulting in better integration of feedback control mechanisms in comparison to a power grip. This would allow for more accurate tactile perception. This could be used to provide better feedback control in the transfer phase, contributing to a smoother handover action. Furthermore, choosing a precision grip has the advantage of covering less of the object’s surface, thus providing more space for the receiver’s free choice of grip. The receiver therefore has a greater choice of possibilities for action, i.e., object affordances (Gibson, 1986). Furthermore, it should be noted that by leaving the object surface free, the receiver has the choice between mirrored and complimentary action (Sartori and Betti, 2015). If exposing object surfaces is a reason for choosing the precision grip, this indicates that the giver is engaged in third-order planning, meaning that they are also considering the subsequent steps that will be executed by the receiver and attempting to ensure a convenient grasp pattern that facilitate the receiver’s subsequent steps (Haggard, 1998).

The hypothesis that the giver considers the receiver’s subsequent actions is further supported by findings which have shown that givers tend to grasp objects at the periphery (instead of at the center of mass) and also tend, when the object has a handle, to leave the handle free and exposed (Cini et al., 2019). This giver behavior, in fact, also offers the receiver the opportunity to freely choose their own grasp pattern.

Another hypothesis that is supported by observing the reach and grasp phase of handover actions is the idea of end-state comfort (Rosenbaum and Jorgensen, 1992; Rosenbaum et al., 1993) and its extension to joint actions (Herbort et al., 2012). To test this hypothesis experimentally, one must again consider the end of handover phase. Manipulating the end of handover phase can modify the receiver’s intention. If this manipulation results in a change in the giver’s behavior, this indicates that the giver is taking the receiver’s end-state comfort into account. This would show that the choice and positioning of the giver’s grasp were not only influenced by the fact that a second person is involved in a joint action (Gonzalez et al., 2011; Meyer et al., 2013; Cini et al., 2019). The results indicate that the type of grasp also depends on the action that the receiver will perform. Although the giver seems to take into account their own end-state comfort as well, the initial grasp action is still performed in such a way that the receiver has the opportunity of beginning and end-state comfort (Gonzalez et al., 2011).

### 4.3. The object transport phase

The handover position and the hand trajectories of the two actors are directly related. The giver’s kinematic in a handover action has strong similarities to a comparable single action task (e.g., replacement). Thus, the velocity profile of the giver’s hand during this phase can be described as the hand accelerating to a certain peak velocity, followed by a deceleration immediately before entering the handover position. This bell-shaped velocity profile is similar to that in the transport phase of a replacement task with accuracy (i.e., a task in which an object is to be placed at a specific location; Sutiphotinun et al., 2020). Thus, the hand trajectory of the giver in the object transport phase is consistent with the minimum jerk theory (Flash and Hogan, 1985). Nevertheless, deviations from single action tasks in the trajectories of the object transport phase could also be shown. This seems to be mainly attributed to a more careful action when the object is handed over to a human than an inanimate, robust container. Extended path and elevation of the wrist trajectory, prolongation of the deceleration phase and lower peak velocity (Becchio et al., 2008) have been shown in human handover actions. These changes in the trajectory of object transport indicates similarities with the changes in a single action task in which subjects were asked to place objects in a fragile container (Marteniuk et al., 1987). Accordingly, this behavior indicates more careful handling in a joint handover action.

While it could be shown that object weight did not influence on the handover position (and thus hand trajectories; Hansen et al., 2017; Sutiphotinun et al., 2020), spatial factors such as the actor’s body size (Sutiphotinun et al., 2020) and distance (Hansen et al., 2017) seemed to affect kinematics in the object transport phase, however, handover height was the only spatial dimension affected. If the distance between the actors is small, it is sufficient for them to mainly use the elbow joint, only moving the shoulder joint enough to reach the handover position. However, the greater the distance between the actors, the more movement of the shoulder joint becomes necessary. The involvement of the shoulder joint presumably results in this increased handover height at greater distances. The actors seem to tend to adjust the handover height to the minimum height of the shared workspace, which of course depends on body size. This would speak in favor of a strategy based on minimal energy consumption (Alexander, 1997). The giver and receiver put a similar amount of effort into the joint handover action while keeping the overall effort minimal. It should be noted, however, that the studies cited here only tested young, healthy adults. It has already been shown that people take into account both environmental and individual constraints of co-actors in joint actions (Schmitz et al., 2017). Thus, if the goal of the actors is to minimize the overall effort of a joint action, a change in handover position should be observed when one of the two actors is constrained in some way. In a handover action between a young, healthy adult person and an adversely hindered person (e.g., toddler, elderly, or physically impaired person), it is to be expected that the handover height would be adjusted to the comfort height of the impaired person. Furthermore, it would be conceivable that the familiarity of the two persons, their gender, and cultural differences may also influence the handover action in the object transport phase. It is known that peripersonal space varies between cultures (Làdavas and Serino, 2010; Brozzoli et al., 2012) and genders (Wabnegger et al., 2016). Accordingly, it can be assumed that the distance for a handover action also differs across cultures, which could be an influencing factor for the actors’ hand trajectories.

In the object transport phase, the coordination of the giver and receiver in time and space plays a major role. A smooth and seamless handover action is only possible if the two actors synchronize properly. Studies have shown that the giver is primarily responsible for the timing in a handover action and that the receiver tends to adjust to the giver in this regard (Mason and Mackenzie, 2005; Sutiphotinun et al., 2020). This means that the receiver, based on the observation of the giver’s kinematics, predicts the position and time at which the object is to be grasped and adapts their own kinematic strategy to it.

Focusing on the giver’s grip forces in this phase of a handover action, showed that these are less accurately matched to the object mass and the inertial force associated with transport than during single action tasks (Mason and Mackenzie, 2005; Endo et al., 2012). Adjustment of grip forces across trials came to different results in the studies depending on whether grip forces could be adjusted and increased (Endo et al., 2012) with repetition of the task (Mason and Mackenzie, 2005). As one study in which grip forces were adjusted over the course of the experiment included a total of 140 trials (Endo et al., 2012) and the other 80 trials (Mason and Mackenzie, 2005), it is possible that the number of trials performed per experiment was decisive for the different results in grip force adjustment. In replacement tasks, it is known that the grip forces are precisely adapted to the fluctuations of the inertia force during the transport phase anticipatorily (Flanagan and Wing, 1993; Nowak, 2004). The absence of this precise anticipatory control in handover actions could be due to the fact that many more factors have to be considered in a handover action than in single action tasks. These additional factors include, for example, anticipating and coordinating the location and timing of the handover with the receiver, and the strength of the collision between the object and the receiver at the end of the object transport phase. Given this complexity, it is conceivable that the number of trials in these studies may not be sufficient to adapt the model as accurately as observed in single action tasks. Another explanation could also be that handover actions are more open and, thus, more variable and less predictable in comparison to single action tasks. It is possible that this reduced predictability makes it impossible to adjust grip forces accurately. Thus, the giver does not even try to execute a precise grip force adjusted throughout the action, rather the giver’s priority is to choose a grip force that is sufficient for all events that may influence the necessary grip forces (e.g., transport of the object, collision between object and receiver). This supports a previously observed task-dependent decoupling of grip and load force (Serrien and Wiesendanger, 2001; Nowak and Hermsdörfer, 2004).

### 4.4. The object transfer phase

The object transfer phase represents the core of a handover action and lasts on average about 500 ms with an object weight of 90 g (Mason and Mackenzie, 2005) or about 640 ms for an object weight of 1.8 kg (Döhring et al., 2020). It begins with the initial contact between object and receiver and ends as soon as the giver disconnects from the object. A smooth and seamless object transfer phase is achieved when the giver and receiver synchronize their rate in change of grip force. This means that from the beginning of this phase, the giver reduces their grip force while the receiver increases their grip force. The results show that although givers have a lower grip force rate of change, the timing of the grip force rate peak is the same for both actors (Mason and Mackenzie, 2005). This suggests that the start of the grip force release is triggered by visual information, i.e., feedforward control is used.

After the collision, haptic feedback is again used to synchronize the grip force scaling (hence grip force rate peaks at the same time). This explanation was tested by manipulating the sensory input of the actors in a handover action. These involved manipulations of haptics (through a glove; Endo et al., 2012; Döhring et al., 2020) as well as restricting visual information (blindfolding; Controzzi et al., 2018; Döhring et al., 2020). In addition, the reaching velocity of the receiver was varied in the object transport phase (Mason and Mackenzie, 2005; Controzzi et al., 2018; Döhring et al., 2020), which affected both feedforward (through visual observation) and feedback mechanisms (through a change in the magnitude of the collision).

The results consistently indicate that the object transfer phase lasts longer when the subjects have no (blindfolded) or little (no movement of the receiver in the object transfer phase (Mason and Mackenzie, 2005)) visual information. This longer duration can be attributed to the fact that there is a delay from the collision to the grip force release that matches the time span for feedback mechanisms (Johansson and Westling, 1984, 1987, 1988a,b). This supports the assumption that the giver’s grip force release is visually triggered and, thus, feedforward controlled.

As the receiver’s reaching velocity does not influence on the timing of the release of grip force in normal vision, it can be concluded that receivers do not make their collision-time prediction by distance-to-contact, but by time-to-contact. This is analogous to catching tasks at varying velocities (Lacquaniti and Maioli, 1989; Savelsbergh et al., 1992). If no visual information is available to the receiver, the coordination of the actors’ movements diminishes and the grip force release must be triggered exclusively by haptic input and, consequently, be feedback-controlled.

When the receiver’s reaching velocity was manipulated in the no-vision condition, this also had an effect on the grip force release. The higher the receiver’s reaching velocity, the shorter the delay until the grip force release began and the higher the grip force rate (Controzzi et al., 2018; Döhring et al., 2020). This response to object-receiver collision is similar to the impulsive catch-up response (Cole and Abbs, 1988; Johansson et al., 1992; Cole and Johansson, 1993). This impulsive catch-up response is indicated by the observation that the greater the collision-induced perturbation, the shorter the delay to the onset of grip force onset and the higher the grip force rate. The effect reversed for the giver in a handover action. This can be explained by the fact that the goal of the giver is to release the object, whereas, in a catch task, the goal is to stabilize the object in the hand. This effect, comparable to the impulsive catch-up response, suggests that the neural system involves a fast feedback mechanism when visual information is missing.

The results of manipulating receiver reaching velocity with normal giver vision showed that givers set their initial grip force release rate by the receiver’s reaching velocity. This suggests that by observing the movement, inferences are made about the receiver’s intention (Kilner et al., 2007; Pfister et al., 2013; Quesque and Coello, 2015; Cavallo et al., 2016; Di Cesare et al., 2016; Quesque et al., 2016; Lelonkiewicz et al., 2020) and the dynamics of the subsequent object transfer phase are derived as a result. These results are consistent with the motor resonance hypothesis, which states that while observing a person’s movements, an internal motor simulation occurs in the brain to interpret that person’s intention and, thus, make a prediction about the following action (Rizzolatti and Craighero, 2004; Springer et al., 2012).

Under normal conditions (no gloves or blindfolds), haptic input was used for feedback control only. Accordingly, in handover actions, haptics was used exclusively for monitoring (Mason and Mackenzie, 2005; Controzzi et al., 2018). This means that the predicted actions of the co-actor are compared with the incoming haptic information and, if necessary, one’s motor planning/execution is adjusted.

To learn more about the relevance of haptic information, experiments were conducted in which gloves were worn (Endo et al., 2012; Döhring et al., 2020). When using gloves in this context, it should always be kept in mind that this manipulation not only affects the haptics but also the frictional properties during the grasping task. It was shown that wearing a glove does not delay the onset of grip force release, which is consistent with the assumption that haptics is used exclusively in feedback control, but grip force release is feedforward controlled. Nevertheless, the duration of the object transfer phase was prolonged by wearing gloves. This could indicate that reducing the amount of haptic information caused uncertainty in the actors’ monitoring process. In one of the studies, generally increased grip forces were found when gloves were worn (Döhring et al., 2020). The reason for this could be that one effect of reduced haptic input is that a larger safety margin is generally required to ensure that the object does not slip. However, there could also be a more general reason for these increased grip forces, namely, the reduced friction between the object and the hand (thus, more force is needed to keep the object from slipping). A prolongation of the object transfer phase was also observed in this experiment (Döhring et al., 2020) and, when the grip force is higher, it can be assumed that the duration of grip force reduction and development would also be longer.

Furthermore, it was also shown that the mass of an object influenced the duration of the object transfer phase (Hansen et al., 2017). The greater the object’s mass, the greater the required grip force. When the grip force rate remains constant in this scenario, it leads to a prolongation of the object transfer phase.

### 4.5. The end of handover

With the onset of the fourth phase, the process of handover itself is completed. During the end of handover phase, the receiver uses the object in line with their intention. Thus, this phase primarily influences the selection of the giver’s grasp pattern and grasp location. As explained in the previous subsections (see Reach and grasp phase), the giver’s assumptions and predictions (and thus knowledge) about the receiver’s intentions influence the giver’s motor planning. If the giver knows what action will be performed in the end of handover phase, this can lead to influencing the giver’s execution of the movement. Hence, this phase can be manipulated to specifically test hypotheses such as engagement in third-order planning.

## 5. Conclusion

This systematic review has demonstrated that only few original studies exist that investigated the kinematic or dynamic characteristics of handover actions in human dyads. In addition, these studies stem from various research disciplines and focus on different research questions. Consequently, a common framework to investigate human handover actions is currently lacking. We have therefore developed such a framework providing a distinct terminology and classification scheme into distinct phases that may be used for future studies. We suggest to differentiate between four phases: (1.) Reach and grasp., (2.) object transport, (3.) object transfer, and (4.) end of handover.

The studies surveyed here have shown that each actor’s action planning and execution are influenced by both knowledge of the co-actor’s intentions and assumptions about their intentions generated through observation of the co-actor. The focus was primarily on the behavior of the givers. It could be shown that givers control their action execution in such a way that the receiver is able to have a comfortable starting position for their planned action. In most studies, although the receiver’s behavior was used as a manipulation, the receiver’s behavior was not the focus of research. Therefore, the question arises whether receivers also adjust their own behavior based on observation of the giver and predictions based on this. To clarify this point, further research is needed.

Furthermore, the results indicate that several concepts known from studies of single action tasks (e.g., replacement) can also be generalized and revisited in the context of joint handover actions. For example, the concept of beginning and end-state comfort is relevant for the entire action sequence and not only at the level of the individual. Action planning also follows the principle of minimum energy consumption for the entire sequence of the handover task, rather than for each individual actor. This should be considered more deeply in further research. It is recommended that handover actions should be studied in dyads with significantly different constraints. Due to the differences between the subjects, the individual activity typically differs in the joint actions, so that the jointly expended energy remains minimal. In contrast, if the individual activity of both actors is not affected by the constraints of one actor, it must be assumed that there is no common concept of action.

Results from the included studies indicate that the grip force release of the giver is feedforward controlled by visual cues and feedback mechanisms are used during the transfer phase to monitor and control the successful transfer of the object. To investigate the role of feedforward and feedback control in more detail, we suggest that further experiments should be conducted in which the availability of sensory input is manipulated. Future studies should also increasingly consider the role of the receiver. In particular, the role of feedforward and feedback control mechanisms on the side of the receiver is poorly understood to date.

## Data availability statement

The original contributions presented in the study are included in the article, further inquiries can be directed to the corresponding author.

## Author contributions

LK designed the concept and layout of the review, with JR and CV-R acting in an advisory capacity. The search formula was proposed by LK and revised and defined together with JR. The literature search and organization was carried out by LK. LK wrote the first draft of the manuscript. JR and CV-R gave repeated feedback on the structure and content of the manuscript. All authors contributed to manuscript revision, read, and approved the submitted version.

## Funding

This research was funded by the Deutsche Forschungsgemeinschaft (DFG, German Research Foundation) – Project-ID 416228727 – SFB 1410, subproject A01.

## Conflict of interest

The authors declare that the research was conducted in the absence of any commercial or financial relationships that could be construed as a potential conflict of interest.

## Publisher’s note

All claims expressed in this article are solely those of the authors and do not necessarily represent those of their affiliated organizations, or those of the publisher, the editors and the reviewers. Any product that may be evaluated in this article, or claim that may be made by its manufacturer, is not guaranteed or endorsed by the publisher.
